# Editorial

**Published:** 1892-11

**Authors:** 


### Editorial.

### SURGICAL RELIEF FOR BILIARY OBSTRUCTION.

The recent improvements in surgical procedures connected with the gall bladder, are among the most notable advances in the operative measures adopted of late for the relief of abdominal troubles.

That system of ducts which connects the liver with the gall-bladder and alimentary canal performs such important functions in health as to render a knowledge of them essential for a proper comprehension of the disorders incident to their temporary arrest or permanent occlusion.

A division of these conduits into the small biliary tubules, the hepatic, cystic and common ducts is generally recognized, and any interruption in their discharge of bile demands correction.

To give surgical relief for biliary obstruction, all the surroundings of the case must be clearly defined. With a view to the proper diagnosis of disorders involving the gall-bladder, all tumors connected with the liver call for careful examination. Any purulent collection in the parenchymatous structure of this organ, extending below the margin of the ribs on the right side, may be mistaken for a distended gall-bladder. On the other hand, the dilated sac may give the impression of hepatic abscess. In the event of the extension of the elongated tumor, from dropsy of the gall-bladder, into the iliac region, it may be taken for an enlargement of the ovary with fluid contents. The element of fluctuation does not avail for a differential diagnosis; but in the absence of any well defined induration around the border of the tumor and its reaching up under the costal arch, it is a fair inference that the gall-bladder is involved. If along with local signs there is evidence of obstruction of the common duct in the appearance of jaundice, or the absence of bile from the fæcal evacuations, it will still further strengthen the presumption in favor of an accumulation, either of bile, a clear mucus, or a sero-purulent fluid in the gall-bladder, and thus contribute towards a correct diagnosis.

In the consideration of different methods of operating on the gall-bladder, it is preferable to commence with that of most gravity and then proceed to others of less seriousness, under the following classification:

Cholecystectomy, or excision of the gall-bladder; cholecystotomy, with or without the formation of an external biliary fistula; duodeno—cholecystostomy, or the formation of a direct communication between the gall-bladder and duodenum.

Cholecystectomy indicates the incision of the walls of the gall-bladder or the extirpation of this sac with ligation of the cystic duct.

The peculiar arrangement of the several ducts by which the bile is conveyed from the secretory apparatus of the liver into the gall-bladder, and thence discharged as occasion may require, may be supplemented by its direct passage. In the event, therefore, of complete occlusion of the cystic duct, while there is no restriction of the direct passage, it becomes utilized without this diversion of the bile into the gall bladder.

A pre-requisite for differentiating the conditions warranting extirpation of the gall-bladder from those derangements in which other modes of correction are available is a thorough comprehension of the normal functions of the gall-bladder, with a minute examination of all the consequences of obstruction of the ducts, and the feasibility of the various measures of relief which have been proposed or put into execution on the human subject.

An incision of the linea alba, between the ensiform cartilage and umbilicus, of sufficient length for convenient manipulation, or over the line of the tumor beneath the arch of the ribs, will expose the diseased gall-bladder, whether it be distended or not. If there is a fluid of any kind accumulated in its cavity, this should be let out by puncture with a trocar, and then incision of the sac as near the margin of the liver as practicably will admit of the removal of any solid contents, with an exploration of the cystic duct. Excision of the walls of the gall-bladder, or a careful dissection of its attachment to the liver, with ligature of the cystic duct if permeable and the subsequent closure of the external wound by suture, completes one of the most rational and effective proceedings in surgery.

We must be guided in our decision of its merits, by the judgment of those whose practical acquaintance with the details of the operation entitles them to recognition as authorities in the matter, and it should not be condemned without a thorough investigation of the grounds upon which it is undertaken. Criticism of this operation from any other standpoint than that herein presented, is short-sighted and unwarrantable, since the facts must be accepted in their true significance, as the basis of forming a correct judgment. The legitimate fruits of an impartial consideration of the cases of extirpation of the gall bladder, which are now brought to light, must soon be apparent with the advance of surgery.

It is clearly shown that the gall-bladder is not indispensable to the regular performance of digestion, and it is not infrequently found completely occluded and atrophied, while the bile is conveyed directly into the duodenum, so that Thiriar regards cholecystectomy as the least dangerous of all the operations.

We had to chronicle eleven additional cases of cholecystotomy shortly after the table of thirty-three cases was published in the Reference Hand-book, there being eight successful cases reported by Tait, two unsuccessful by Parks, and a successful case for each by Carmalt and Hutchison. All these operations were done by attaching the opening in the gall-bladder to the wound in the parietes, so as to allow of drainage externally, with a view to a temporary biliary fistula, but expecting their closure subsequently. Quite a large number of these cases have been reported within the past few years.

The complete success of Tait's operations upon this principle, and the satisfactory results of attaching the vesical incision to the external wound in the hands of a number of other operators, has to a large extent redeemed this measure from the imputation of entailing upon the patient the grave consequences of a continued drain of bile from the system. In cases, therefore, which afford a reasonable prospect of a reestablishment of the flow of bile into the duodenum, this means of giving an outlet externally to the contents of the gall-bladder, until obstructions of an organic or mechanical nature may disappear, commends itself to the adoption of surgeons, but it is not suited to cases of complete occlusions.

Duodeno Cholecystostomy. This term indicates an operation for uniting the gall-bladder and duodenum, with an opening between them for the passage of the bile. It is only applicable in cases of permanent occlusion of the common bile-duct, while the cystic duct is permeable, or may be opened by surgical means, and the sac has not undergone degeneration.

If there is satisfactory evidence of such agglutination of the walls of the common duct as to render this canal incapable of conveying the bile into the alimentary tube, we may undertake to substitute an abnormal for the normal route of the bile, by a direct cysto-duodenal communication. This is not a circuitous channel from the liver into the intestine, since a large portion of bile flows ordinarily into the gall-bladder, and by this new outlet it will pass immediately into the duodenum. The operation proposed by Gaston for effecting a union between the walls of the gall-bladder and duodenum, with an opening from the cavity of the former into that of the latter, consists in connecting them by suture with a single stitch, which shall unite their surfaces by adhesive inflammation and cut an opening between them, passing off in the intestinal canal. Others have used different processes since.

The prime consideration in determining upon a resort to cholecystectomy, cholecystotomy or to duodeno-cholecystostomy is the comparitive integrity of the structures involved in disease of the gall-bladder from biliary obstruction. If there should exist such an impairment of the vitality as to threaten disorganization of the tissues of the sac, excision or extirpation would be preferable to any operation for preserving an outlet for the bile, either by an external opening or by a communication with the duodenum. If the conditions warrant a conclusion that the normal communication between the gall-bladder and the alimenary canal can be restored, cholecystotomy would be justifiable. But in case of permanent obstruction of the common bile-duct, without such obstruction of the cystic duct as to afford an irremediable impediment to the entrance of bile into the gall-bladder, duodeno-cholecystostomy is the most feasible proceeding. Permanent occlusion of common duct warrants a direct outlet from the gall-bladder into the duodenum. In the event of an apparent obstruction of the cystic duct, it is usually complicated with accumulations within the sac, of inspissated bile, of mucus, of pus, of sero-purulent fluid or of gall-stones. The removal of the contents of the gall-bladder and the impediment from the duct, devolves upon the surgeon before undertaking to connect the gall-bladder with the duodenum. This may be done by an incision of limited extent, so that it shall be sutured to an incision of the duodenum or the jejunum and thus secure their union and the passage of the bile into the intestine instead of being discharged externally. It is proper under other circumstances to evacuate the distended gall-bladder externally, and suture the incision separately with a view to establish another communication between it and the canal, which is requisite for a successful result in any case.

When the diagnosis of distension of the gall-bladder can be made out, as most frequently it can, upon a thorough manual examination connected with the history of biliary obstruction, it is preferable that an abdominal incision be resorted to as a preliminary step to whatever exploration by the eye, hand or instruments may be requisite for completing the operation. Should it be found practicable to free the tract of the cystic duct while the common duct remains impermeable, the proper recourse consists in a direct cholecysto-duodenal outlet for the bile into the alimentary canal.

A number of operations have been performed in which the gall-bladder has been connected with the small intestine and the colon. The most satisfactory results however, have been secured by the union with the duodenum.

The common bile duct has been united to the intestine in a few cases, but this operation is attended with difficulties which are not encountered in effecting a communication with the gall-bladder.

The removal of the biliary calculi from the ducts by incision of the walls and then closing the opening by suture, has also been resorted to for freeing the canal of their obstructions.

ON THE afternoon of October 25th, at three o'clock, Dr. L. P. Stephens was married to Miss Mary Bell, at the First Methodist Church, this city. Dr. Stephens is a young man who is held in high esteem by the profession, and to him and his fair young bride THE RECORD tenders its heartiest congratulations.

---

DR. DAN H. HOWELL, business manager of THE RECORD, has been absent for the past three weeks on a business and pleasure trip to New York and other Northern cities.

---

WEDNESDAY evening, November 2nd, Miss Eloise Gaston, daughter of Dr. J. McFadden Gaston, will be wedded to Mr. Thos. Bolling Gay, at the First Presbyterian Church. Miss Gaston inherits many of the characteristics of her distinguished father, and is a young lady of superior intellect and rare accomplishments. Mr. Gay has just reason to be proud of the prize he will have won.

---

DR. C. D. HURT, one of Columbus' foremost citizens, has come to Atlanta to practice his profession. We welcome him to our midst.

---

DR. A. T. ROWE, of Columbiana, Ala., has recently moved to Atlanta and has located at West End. The doctor is a man of brilliant intellect and thorough medical training, and what the profession of Alabama has lost has been our gain.

---

ON THE evening of the 20th of October, Miss Nora Earnest, daughter of Dr. J. G. Earnest, was married to Mr. Charlie Northen, nephew of Gov. W. J. Northen. The ceremony was performed at the First Presbyterian Church, and a reception tendered them at the Executive Mansion. THE RECORD extends its congratulations.

---

DR. A. G. HOBBS has resigned the chair of Diseases of Eye, Ear and Throat in the Southern Medical College.

---

WE REGRET our inability to publish a London letter this month, because of its late arrival. We hope in future to be able to give it to our readers regularly.

